# Three muscle slings of the pelvic floor in women: an anatomic study

**DOI:** 10.1007/s12565-019-00492-4

**Published:** 2019-06-04

**Authors:** Phichaya Baramee, Satoru Muro, Janyaruk Suriyut, Masayo Harada, Keiichi Akita

**Affiliations:** Department of Clinical Anatomy, Tokyo Medical and Dental University (TMDU), 1-5-45 Yushima, Bunkyo-ku, Tokyo, 113-8510 Japan

**Keywords:** External anal sphincter, Levator ani, Pelvic floor, Perineal muscle, Superficial transverse perineal muscle

## Abstract

The region anterior to the anal canal in women is composed of intertwined smooth and skeletal muscles. The present study aimed to clarify skeletal muscle morphology in the anterior region of the anal canal. The pelvic floor muscles of 28 pelvic halves from 16 female cadavers (mean age 79.75 years) were dissected from the inferior aspect to examine the perineal muscles, followed by midline transection and dissection from the inner surface to examine the pelvic outlet muscles. The bulbospongiosus muscle was found to be attached to the lateral surface of the external anal sphincter. The superficial transverse perineal muscle crossed superiorly to the bulbospongiosus and coursed medially toward its contralateral muscle bundle deep to the anterior portion of the external anal sphincter. The superficial transverse perineal muscle formed the middle sling. From the medial aspect, the anterior part of the levator ani was divided into anterior and posterior bundles to form the anterior and posterior slings, respectively. This study proposes that three muscular slings could be important in supporting the pelvic floor in women. In addition, this study shows that the anterior skeletal muscular wall of the anal canal is composed of the anterior muscle bundle of the levator ani, superficial transverse perineal, and proper external anal sphincter muscles.

## Introduction

The normal complex anatomy of the pelvic floor in women has been discussed extensively, especially with reference to the perineal body (DeLancey 1999; Petros 2001; Shafik et al. 2007; Soga et al. 2007; Abendstein et al. 2008; Stein and DeLancey 2008; Larson et al. 2010; Santoro et al. 2015). It is generally accepted that most of the perineal skeletal muscles are attached to the perineal body. In women, the longitudinal and circular layers of the smooth muscle are believed to be in the anorectal anterior wall, and the perineal body is described as a fibromuscular mass located between the anorectal canal and the vagina (Woodman and Graney 2002; Wu et al. 2015; Plochocki et al. 2016). However, Muro et al. (2019) recently described that smooth muscle fibers of the circular muscle (internal anal sphincter) and the longitudinal muscle extend anteriorly to occupy the region of the perineal body in women. Therefore, the perineal body may not be an independent tissue, and these tissues could be separated from the skeletal muscle bundles.

Previous studies investigating the positional relationships among the bulbospongiosus muscle (BS), the superficial transverse perineal muscle (STP), and the external anal sphincter (EAS) reported that these three muscles were continuous anatomically (Plochocki et al. 2016), and demonstrated the relationships among the pelvic outlet muscles in three-dimensional (3D) topography (Larson et al. 2010; Wu et al. 2015). However, the positional relationships between the STP and the EAS remain unclear and controversial.

We have previously examined the histological characteristics of the pelvic floor, especially the distribution and positional relationship of the skeletal and smooth muscle tissues, in embalmed cadaveric studies (Muro et al. 2014, 2018, 2019; Tsukada et al. 2016; Nakajima et al. 2017). We reported that the pelvic floor is composed mainly of smooth and skeletal muscles, which are interconnected in a complex manner, especially in the region just anterior to the anal canal. Moreover, we found that the smooth and skeletal muscle areas were well identified.

In the present study involving female cadavers, we attempted to clarify the morphology of the skeletal muscles in supporting the pelvic floor. We aimed to carefully dissect the smooth muscle in the pelvic floor to preserve the skeletal muscle. In addition, we compared our findings with the previous study to understand the anatomical relationship between the STP and the EAS (Larson et al. 2010; Wu et al. 2015).

## Materials and methods

All cadavers used in this study were obtained from the dissection room of our institute. The cadavers were donated to the department of anatomy for use in anatomical studies in accordance with the guidelines of the Act on Body Donation for Medical and Dental Education law of Japan. All cadavers used in this study were fixed by arterial perfusion with 8% formalin and preserved in 30% alcohol to prevent fungal growth and maintain tissue softness.

### Macroscopic anatomical investigation

A total of 28 pelvic halves from 16 female cadavers (age range 54–99 years, mean age 79.75 years) were used for dissection. Four pelvic halves were not used in this research because they were destroyed during dissection and it was not possible to observe the muscle fiber clearly. In all specimens, the pelvic regions were obtained en bloc from the cadavers. The muscles of the pelvic floor were dissected from the inferior aspect. The perineal muscles, including the ischiocavernosus (IC), the BS, and the STP, were carefully dissected to examine the muscle bundles and the relationship between them. In addition, the pelvis was dissected from the ischioanal fossa to investigate the EAS and levator ani (LA). To observe the muscle bundles in the inner surfaces of the pelvic outlet muscles, the pelvis was transected in the midline, and internal organs, adjacent connective tissues, and smooth muscle tissues were meticulously removed.

## Results

### Lateral and inferior aspects

**Fig. 1:**
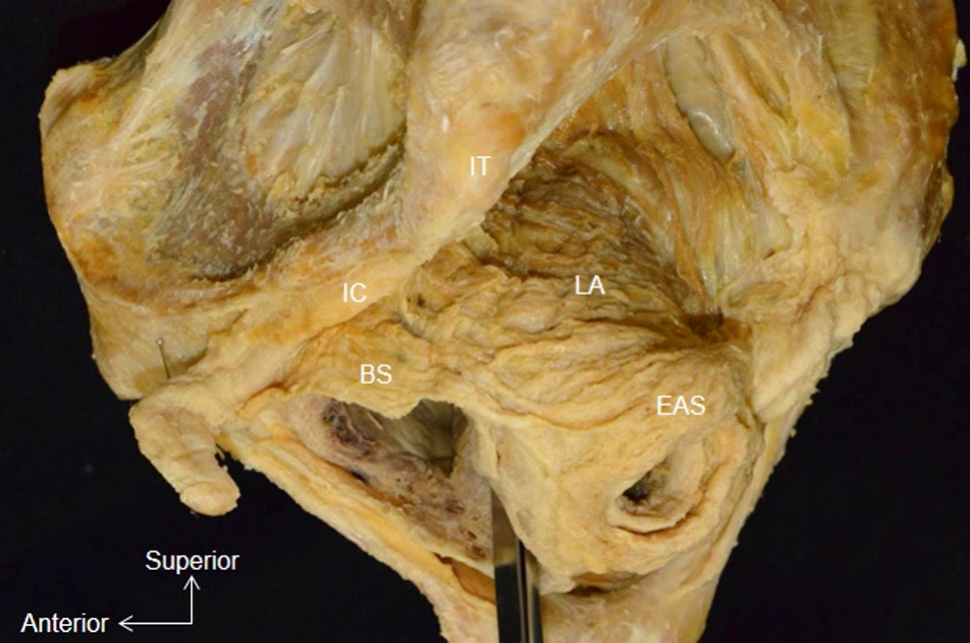
Whole pelvis dissection, lateral aspect. The muscle bundle of the bulbospongiosus is attached to the lateral surface of the external anal sphincter. *BS* Bulbospongiosus, *EAS* external anal sphincter, *IC* ischiocavernosus, *IT* ischial tuberosity, *LA* levator ani

From the lateral aspect, the BS was found to be attached anteriorly to the corpus cavernosus of the clitoris, and its muscle bundles extended posteriorly to cover the bulb of the vestibule. In addition, the BS had an attachment to the lateral surface of the EAS (Fig. 1).

**Fig. 2:**
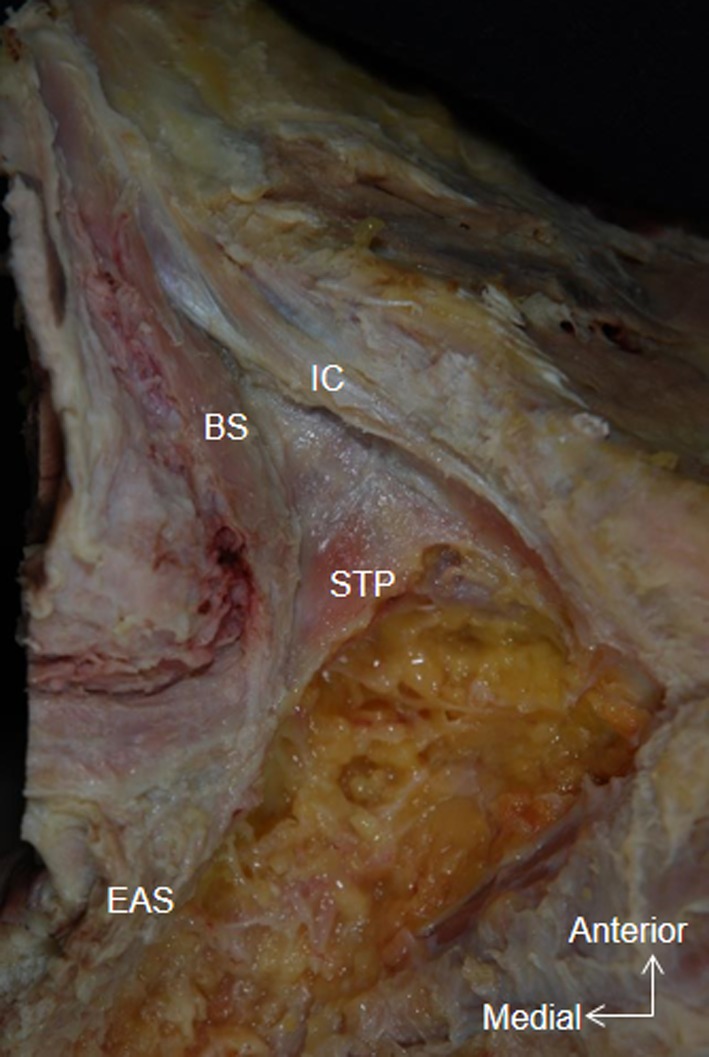
Whole pelvis dissection, inferior aspect. The muscle bundle of the BS fuses with the lateral surface of the EAS. The superficial transverse perineal muscle crosses the BS superiorly. *STP* Superficial transverse perineal muscle

**Fig. 3:**
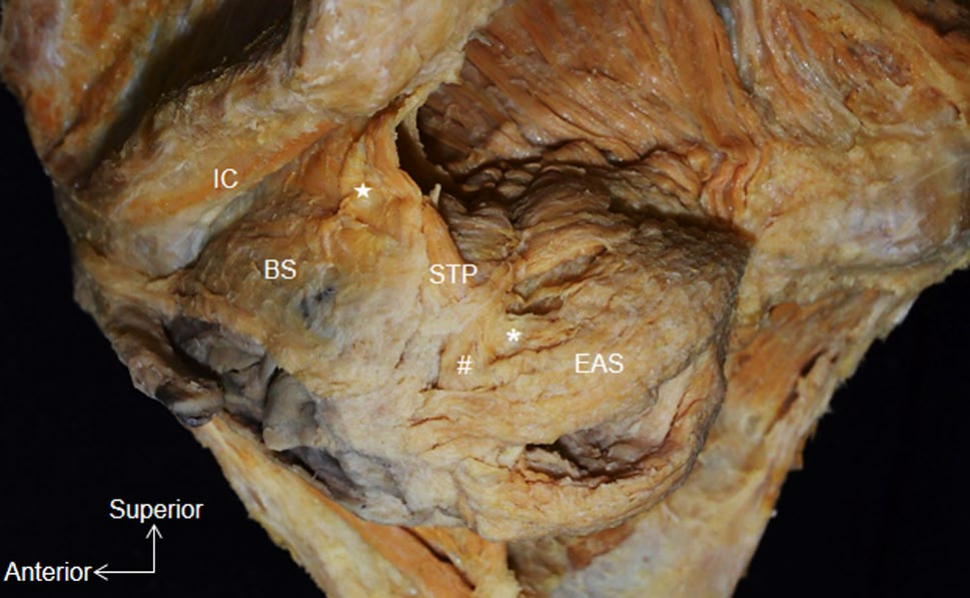
Lateral aspect of pelvic dissection, right side (mirror image). A few muscle bundles occupy the region between the BS and the STP (*star*). The STP extends posteriorly along the external anal sphincter (*asterisk*). The medial part of the STP courses medially along the inner surface of the EAS (*hash symbol*)

The STP originated from the ischial tuberosity, extended inferomedially, and crossed superior to the BS (Fig. 2). The medial part of the STP extended over the inner surface of the EAS (indicated by a hash sign in Fig. 3). In addition, some muscle bundles extended posteriorly along the lateral surface of the EAS (indicated by an asterisk in Fig. 3). A few muscle bundles were also observed in the region between the BS and the STP (indicated by a star in Fig. 3).

**Fig. 4:**
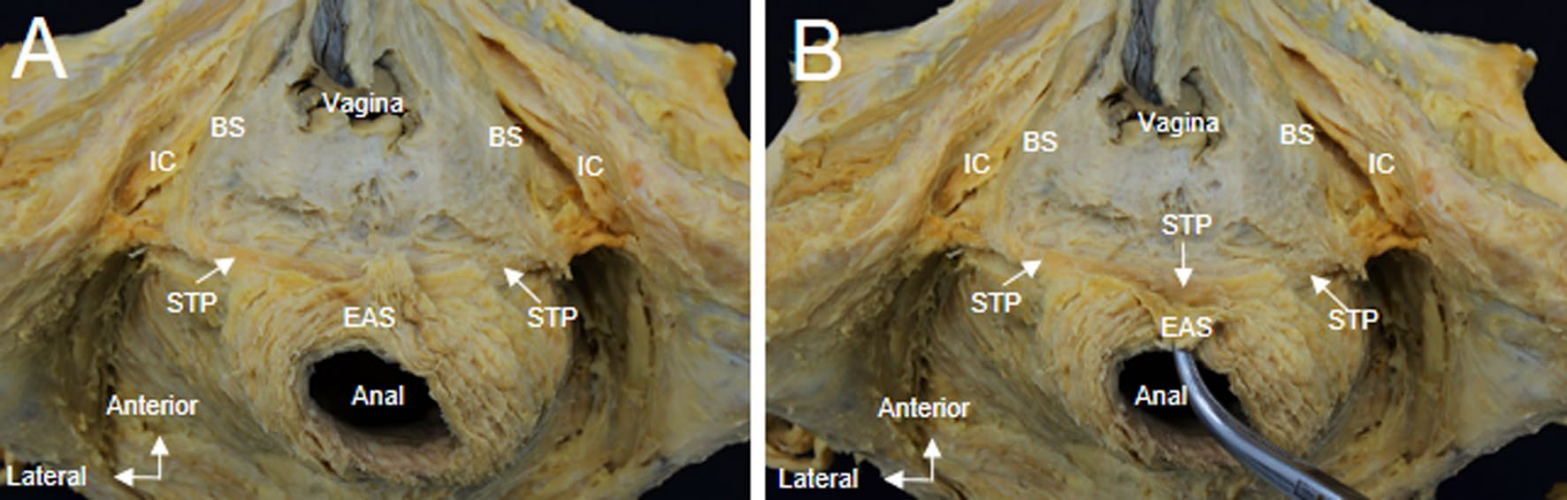
**a** Whole pelvis dissection, anteroinferior aspect. The anterior portion of the EAS covers the middle part of the STP. **b** On reflection of the anterior part of the EAS, the STP is identified crossing the midline and continuing as the same muscle on the contralateral side

In the anteroinferior aspect, bundles of the STP extended medially and were covered by the anterior portion of the EAS (Fig. 4a). After reflection of the anterior part of the EAS, the STP was observed to fuse with the contralateral muscle bundle (Fig. 4b).

### Medial aspect

**Fig. 5:**
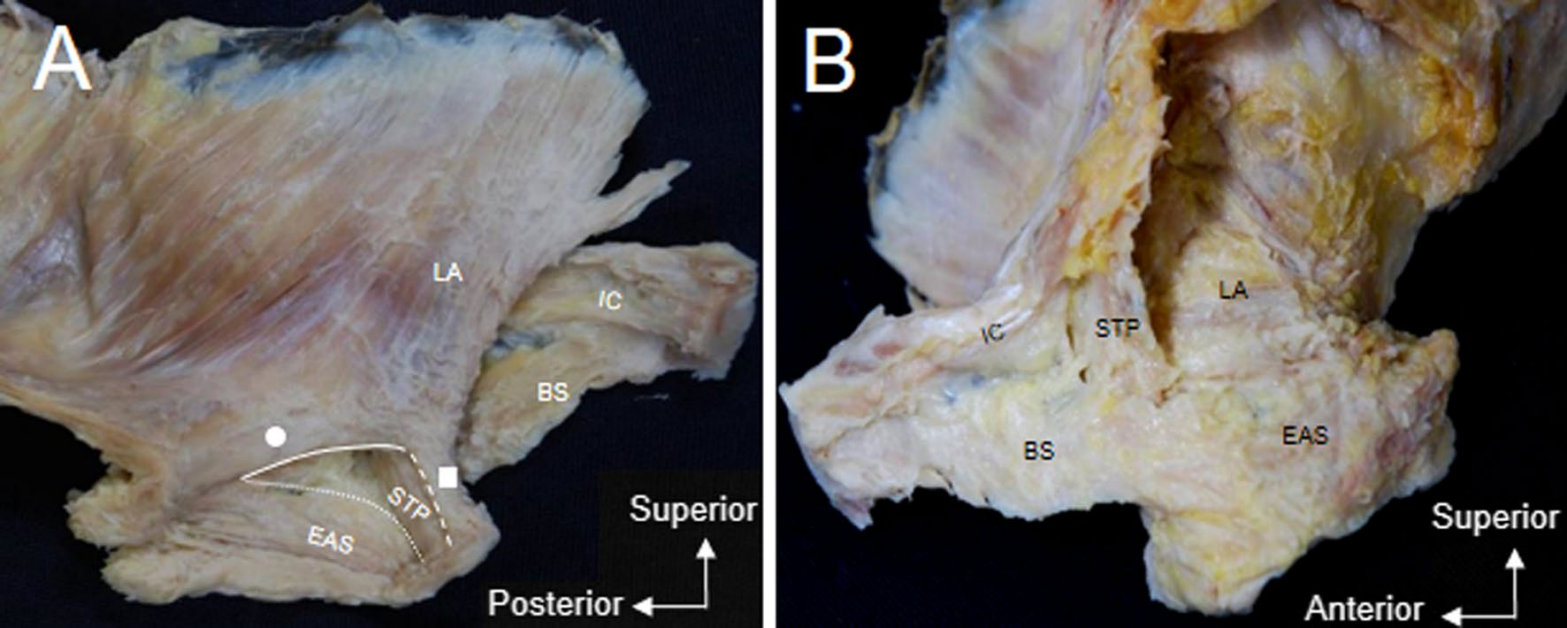
**a** Medial aspect of pelvic dissection, left side. The anterior bundle (*square*) and the posterior bundle (*circle*) of the anterior part of LA are seen superior to the EAS. The inferior border of the anterior bundle of the anterior part of the LA (*dashed line*) and the posterior bundle of the anterior part of the LA (*solid line*) and the superior border of the EAS (*curved line *) form a triangular space. The STP passes through this triangular space and courses along the inner surface of the EAS. **b** Lateral aspect of the same specimen, left side. The STP extends superomedial to the posterior part of the BS, along the inner surface of the EAS

From the medial aspect, the BS was observed to extend posteriorly and lie lateral to the EAS (Fig. 5a). The anterior part of the LA originated from the posterior aspect of the body of the pubis and extended posteriorly. The anterior part was divided into two bundles: (1) the posterior bundle (indicated by a circle in Fig. 5a) extended posteriorly to surround the posterior anal region and along with the contralateral muscle bundles formed the posterior sling, and (2) the anterior bundle (indicated by the square in Fig. 5a) extended medially to surround the anterior anal region and along with the contralateral muscle bundle formed the anterior sling. Therefore, the anterior part of the LA from either side formed a loop around the anal canal, superior to the EAS.

A triangular space was identified between the inferior borders of the anterior and posterior slings and the superior border of the EAS (indicated by the three lines in Fig. 5a). This space was traversed by the STP, which passed from the ischioanal fossa onto the inner surface of the EAS (Fig. 5) to fuse with its contralateral muscle bundles and form a muscle bundle sling in the anterior part of the anal region. Based on its positional relationship with the other muscle slings, this muscle sling may be referred to as the middle sling.

The anterior muscular wall surrounding the anal canal was composed of the anterior bundle of the anterior part of the LA, STP, and EAS. The anterior bundle of the anterior part of the LA extended superiorly to cover the STP, which was located on the inner surface of the EAS. The anterior bundle, as well as the STP, connected with their contralateral muscle bundles. Therefore, the EAS was not the only component of the anterior wall of the anal canal but was supplemented by the two slings of the anterior bundle of the anterior part of the LA and the STP.

## Discussion

The present study examined the interconnected muscle bundles of the main muscles of the female pelvic outlet in detail. Our main findings were as follows: (1) the BS attaches to the lateral surface of EAS, (2) the STP crosses superiorly to the BS and extends to the inner surface of the EAS before fusing with its contralateral muscle bundle, and (3) the anterior part of the LA is composed of two parts, i.e., the anterior bundle, which extends medially to form the anterior sling; and the posterior bundle, which extends posteromedially to form the posterior sling. In addition, many tiny connecting muscle bundles among the main muscles were observed. However, such tiny muscle bundles have already been described in detail in several reports (Henle 1866; Plochocki et al. 2016). In the present study, we focused on our three main findings.

### Posterior attachment of the BS

The BS is generally described as being attached to the perineal body (Standring 2016). However, whether the BS attaches to the perineal body or extends over it is unclear. In an anatomical study, Shafik et al. (2005, 2007) described fibers of the EAS as extending across the perineal body to form the BS. Mittal et al. (2014) used ultrasound images and magnetic resonance imaging (MRI) based muscle fiber tracking and identified that the EAS fibers cross over from one side to the other in the perineal body and continue as the BS. Arakawa et al. (2010), based on embryological development, proposed that the BS extends posteriorly to communicate with the EAS. Santoro et al. (2015) stated that the BS attaches to the lateral surface of the perineal body and does not meet its contralateral muscle. Recently, Plochocki et al. (2016), in an anatomical study, reported that the BS coursed posteriorly to form the EAS. Their findings are similar to those of Larson et al. (2010), which were based on 3D reconstruction of MR images, and the findings of Wu et al. (2015), which were based on 3D reconstruction of serial anatomical sections. Our findings suggest the presence of a lateral connection between the BS and the EAS. Therefore, in women, the BS independently attaches to the lateral surface of the EAS.

Muro et al. (2019) stated that the perineal body in women may be closely related to the longitudinal smooth muscle layer of the rectum. They reported that, in the anorectal anterior wall, the smooth muscle fibers of the longitudinal muscle converged to the median region and extended anteriorly to become a component of the perineal body. In addition, since the female perineal body occupies a large area (Larson et al. 2010; Wu et al. 2015; Muro et al. 2019), it is anatomically reasonable that both sides of the BS are separated by the perineal body.

### Anterior part of the LA

Kearney et al. (2004) reviewed and organized various terms used in numerous reports about the LA. The LA is generally described in textbooks as being composed of the pubococcygeus, puborectalis, and iliococcygeus (Federative Committee on Anatomical Terminology 2009; Standring 2016). Kearney et al. (2004) pointed out that the term pubococcygeus incorrectly implies a connection between the pubis and coccyx since most of the muscle inserts into the walls of the vagina and anorectum to elevate these structures and close the genital hiatus. They supported the term pubovisceral, as described by Lawson (1974), instead of pubococcygeus.

In the present study, we observed that the anterior part of the LA, which originates from the pubis, is divided mainly into anterior and posterior muscle bundles. Tsukada et al. (2016) reported the direct attachment of the superficial layer of the LA to the longitudinal muscle layer of the rectum. Although the superficial layer could be considered a part of the pubovisceral muscle, the authors focused on the anterior and posterior muscle bundles that form the anterior and posterior slings. Ayoub (1979) classified the anterior portion of the LA into three muscle layers, namely, pelvic, middle, and perineal layers, and stated that a muscle bundle of the perineal layer unites with that of the opposite side in the front of the lower part of the anal canal. This muscle bundle may be similar to the puboprerectalis muscle described by Oh and Kark (1973). However, in the terms compiled by Kearney et al. (2004), the anterior sling is not explained. Uchimoto et al. (2007), in a histological study, reported that the muscle bundles of the bilateral LA attach to the superior surface of the EAS in men, and that the sling of the LA did not encircle the anorectum anteriorly. As Nakajima et al. (2017) found in male pelvises, Muro et al. (2018, 2019) reported that the region just anterior to the anal canal involved a complex interconnection between smooth and skeletal muscles. In the present study, since we carefully removed all structures other than the skeletal muscle bundles, we were convinced that we could clearly dissect the anterior muscle bundle anterior sling.

Holl (1897) defined puborectalis as a muscle bundle loop enclosing the lower part of the rectum. Generally, the puborectalis forms a U-shaped sling posterior to the anal canal (Fritsch 2007; Stoker 2009; Wu et al. 2015; Standring 2016). The posterior muscle bundle of the anterior part of the LA observed in the present study is composed mainly of the puborectalis and forms the posterior sling as previously described (Holl 1897; Fritsch 2007; Stoker 2009; Wu et al. 2015; Standring 2016).

### STP crossing the midline

The STP are generally described as originating at the ischial tuberosities and inserting at the lateral surface of the perineal body (Standring 2016). However, there are several alternate descriptions of this muscle in women. Shafik et al. (2005) described that the STP forms a tendinous fiber close to the midline and decussates with its counterpart from the opposite side. Based on MRI findings, Stoker (2009) found that STP lies superior to the EAS, often with some overlap. Santoro et al. (2015) performed an endovaginal ultrasonography (US) and observed that the STP attaches to the lateral surface of the perineal body along with the EAS and the BS.

Oh and Kark (1973) found that the STP crosses the midline and gives fibers to the EAS on the other side. Based on 3D US images and MRI studies in women, Mittal et al. (2014) proposed a “purse-string” morphology to describe the EAS crossing over to continue as contralateral transverse perineal muscle. Based on 3D reconstruction images from MRI, Larson et al. (2010) demonstrated that the STP traverses the perineal body and extends laterally across the midline anterior to the EAS. Wu et al. (2015), based on 3D reconstruction of serial anatomical sections, identified the STP as being covered by the EAS and extending to the contralateral side. These findings demonstrate that the STP is not directly attached to the perineal body.

In the present study, we found that the STP crossed the midline and continued as the same muscle to the contralateral side. In addition, the STP coursed on the inner surface of the EAS to form the middle sling. The skeletal muscular wall anterior to the anal canal in women is composed of the anterior muscle bundle of the LA, EAS, and STP. Nakajima et al. (2017) dissected the anterior muscular wall of the anal canal in male cadavers and found that the STP does not course on the inner surface of the EAS. Muro et al. (2019) described that the anterolateral longitudinal muscle bundles curve to the midline and fuse with each other anterior to the lowest region of the anal canal in women. This corresponds to the present study, which found that the skeletal muscular walls anterior to the anal canal in women are connected with each other in the midline.

### Three muscle slings of the pelvic floor

**Fig. 6:**
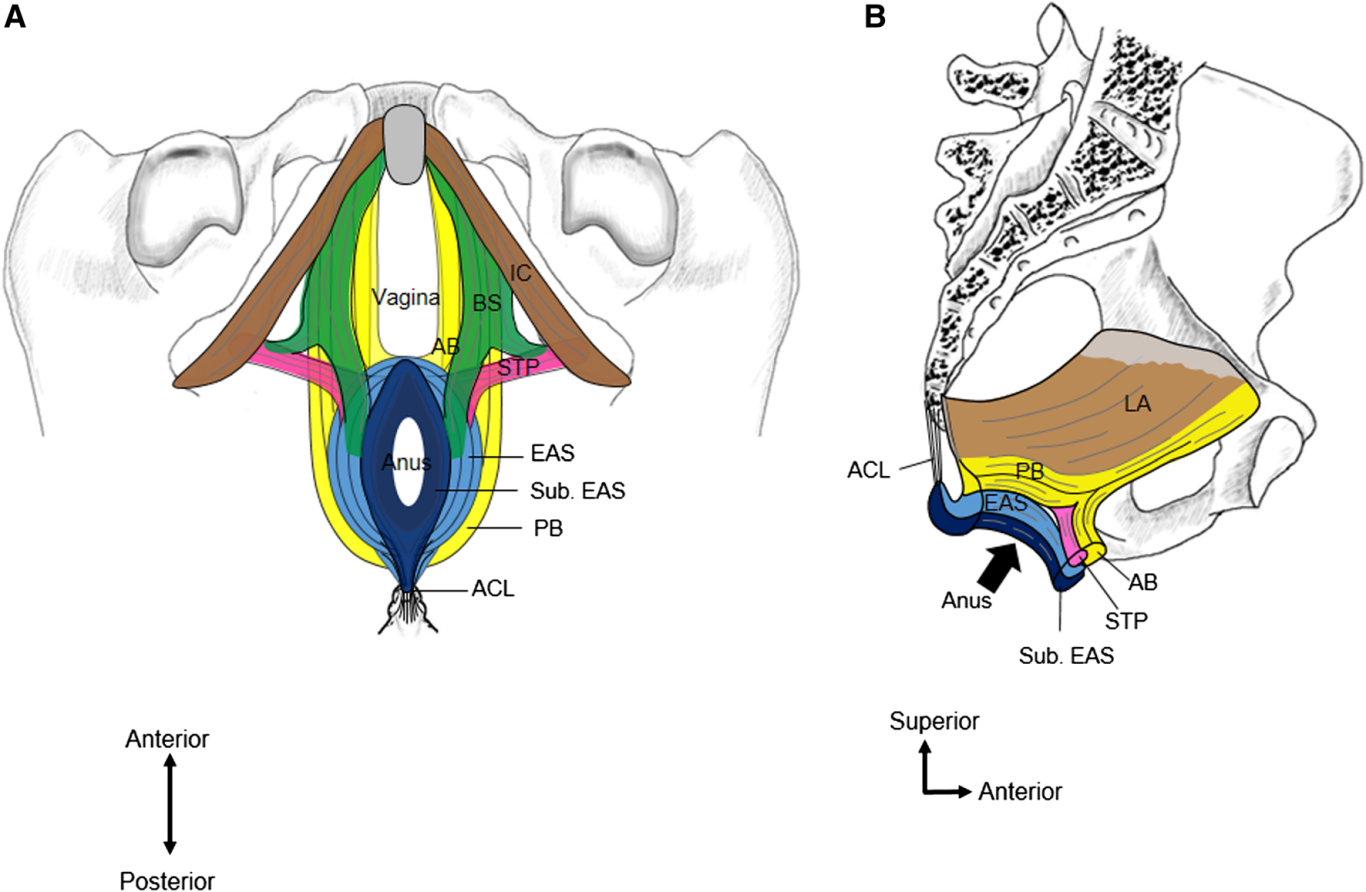
**a** Schematic representation of the three muscle slings in the pelvic floor shown from inferior aspect. **b** The anterior muscular wall of the anal canal from medial aspect. *AB* Anterior bundle of the anterior part of the LA, *ACL* anococcygeal ligament, *BS* bulbospongiosus, *EAS* external anal sphincter, *LA* levator ani, *PB* the posterior bundle of the anterior part of the LA, *STP* superficial transverse perineal muscle, *Sub. EAS* subcutaneous part of the EAS

Figure 6 provides a schematic illustration of the anatomical relationship between the BS, EAS, anterior muscle bundle of the LA, posterior muscle bundle of the LA, and STP, based on our results. According to our findings, the pelvic floor muscles have three muscular slings: the anterior sling formed by the anterior muscle bundle of the LA, a middle sling by the STP, and a posterior sling formed by the posterior muscle bundle of the LA. Muro et al. (2014) described that the posterior muscle bundles of the EAS combine to attach to the anococcygeal ligament inferior to the coccyx and posterior to the EAS. Therefore, these three muscular slings and the anococcygeal ligament may support the pelvic floor in women, and prevent pelvic organ prolapse.
